# SenseRisc: An instrumented smart shirt for risk prevention in the workplace

**DOI:** 10.1017/wtc.2025.10

**Published:** 2025-05-02

**Authors:** Christian Tamantini, Fabrizio Marra, Joshua Di Tocco, Stefano Di Modica, Antonio Lanata, Francesca Cordella, Maurizio Ferrarin, Francesco Rizzo, Mara Stefanelli, Maddalena Papacchini, Corrado Delle Site, Alessio Tamburrano, Carlo Massaroni, Emiliano Schena, Loredana Zollo, Maria Sabrina Sarto

**Affiliations:** 1 Institute of Cognitive Sciences and Technologies, National Research Council of Italy, Rome, Italy; 2 Unit of Advanced Robotics and Human-Centred Technologies, Departmental Faculty of Engineering, Università Campus Bio-Medico di Roma, Rome, Italy; 3 Department of Astronautics, Electrical and Energy Engineering (DIAEE), University of Roma La Sapienza, Rome, Italy; 4 Research Center on Nanotechnologies Applied to Engineering (CNIS), University of Roma La Sapienza, Rome, Italy; 5 Unit of Measurements and Biomedical Instrumentation, Departmental Faculty of Engineering, Università Campus Bio-Medico di Roma, Rome, Italy; 6 Intecs s.p.a, Rome, Italy; 7 Department of Information Engineering, University of Florence, Florence, Italy; 8 Fondazione Policlinico Universitario Campus Bio-Medico, Rome, Italy; 9 IRCCS Fondazione Don Carlo Gnocchi Onlus, Milan, Italy; 10 Department of Technological Innovations and Safety of Plants, Products and Anthropic Settlements (DITSIPIA), National Institute for Insurance against Accidents at Work (INAIL), Rome, Italy

**Keywords:** wearable system, cloud computing, user state estimation, graphene sensors

## Abstract

The integration of wearable smart garments with multiple sensors has gained momentum, enabling real-time monitoring of users’ vital parameters across various domains. This study presents the development and validation of an instrumented smart shirt for risk prevention in workplaces designed to enhance worker safety and well-being in occupational settings. The proposed smart shirt is equipped with sensors for collecting electrocardiogram, respiratory waveform, and acceleration data, with signal conditioning electronics and Bluetooth transmission to the mobile application. The mobile application sends the data to the cloud platform for subsequent Preventive Risk Index (PRI) extraction. The proposed SenseRisc system was validated with eight healthy participants during the execution of different physically exerting activities to assess the capability of the system to capture physiological parameters and estimate the PRI of the worker, and user subjective perception of the instrumented intelligent shirt.

## 1. Introduction

In the context of occupational environments, workers face various risks that can lead to injuries and illnesses. According to the International Labour Organisation (ILO), nearly 3 million people are estimated to have died from work-related injuries and illnesses in 2019, with the majority of cases being occupational diseases (89%) and around 11% resulting from workplace accidents. Additionally, 395 million workers suffered non-fatal accidents, imposing a significant burden on families and society (Podgorski et al., 2017; Maguire et al., 2023). This underscores the critical need for innovative technological solutions that focus on enhancing the health and safety of workers in the workplace (Forat et al., 2021; Nnaji et al., 2021; Fanti et al., 2022).

One of the key advancements in occupational health and safety is the development of intelligent personal protective equipment (Basodan et al., 2021; Santos et al., 2022). This technology leverages miniaturized electronics to monitor physiological parameters, offering innovative solutions across healthcare, telemedicine, sports, and occupational safety sectors (Viegas et al., 2016). Notably, smart shirts have emerged as a significant tool for health and fitness monitoring (Khundaqji et al., 2020). These garments can collect multimodal data from users, including environmental, physiological, and motor parameters, and transmit it to mobile devices or computers for comprehensive analysis (Ometov et al., 2021; Yadav et al., 2023).

Numerous smart clothes have been developed for health monitoring, such as the PSYCHE project for bipolar disorder (Paradiso et al., 2010), AccYouRate for detecting health conditions (Neri et al., 2023), and Hexoskin for cardiac and respiratory monitoring (Montes et al., 2018). While many smart garments like these have been tested for physiological monitoring in various activities (Farjadian et al., 2013; Tada et al., 2015; D’Abbondanza et al., 2021; Nigusse et al., 2021), they are not specifically designed for occupational health and safety, presenting a significant opportunity for targeted technological advancements in this field.

In occupational settings, the continuous monitoring of key physiological parameters such as heart rate and respiratory rate is increasingly recognized as a valuable strategy for preventing work-related risks (Tamantini et al., 2023). These indicators reflect both the worker’s current health status and the physiological load imposed by specific tasks. When combined with movement data, these parameters enable a contextualized interpretation of physical strain, which is essential for assessing the real-time risk associated with various job activities.

Several wearable systems have been proposed in the literature to address safety monitoring in hazardous work environments. For instance, the prototypes presented in Lage et al. (2015) and Hinze et al. (2022) were specifically developed for firefighters and forestry workers, integrating sensors for ECG and galvanic skin response. While effective in capturing basic physiological signals, these systems lack intelligent data processing or real-time feedback mechanisms for the user. A similar limitation is found in the smart shirt introduced in Catarinucci et al. (2022); Šolić et al. (2022), which focuses on thermal stress monitoring through integrated skin temperature sensors, photoplethysmography, and inertial measurement units. These systems are designed for deployment in operational contexts and can provide thermal warnings, yet they do not include respiratory monitoring—an essential parameter for more comprehensive risk estimation.

In addition to textile-based solutions, wearable devices such as the multisensory bracelet proposed in Márquez-Sánchez et al. (2021) allow the monitoring of heart rate and skin temperature in high-risk environments. Likewise, the system developed in De Fazio et al. (2022) extends monitoring capabilities to environmental metrics, including gas concentrations and oxygen levels, making it suitable for hazardous industrial contexts. Finally, efforts to integrate respiratory monitoring into wearable garments have been explored in Mannée et al. (2020), where a fabric-based sensor was tested on an artificial torso. However, this prototype has yet to be validated in real working scenarios, highlighting the gap that still exists between laboratory feasibility and field applicability.

Although existing studies have demonstrated the viability of monitoring devices integrated into smart garments, they have also revealed shortcomings in several crucial aspects. It is notable that there is a dearth of experimental validation, including the use of different subjects, which would assess the smart garment across a range of conditions. Furthermore, these studies do not explore user perception, particularly how users feel about wearing these devices over time, which is crucial for their long-term adoption in occupational settings. Additionally, the scope of monitoring in these studies is often limited to a single derived parameter, for example, thermal stress, which does not provide a holistic view of a worker’s health or safety status. This narrow focus overlooks other potential risk factors that could be critical for ensuring comprehensive occupational safety and health monitoring.

The objective of this contribution is to present the SenseRisc system, an innovative smart shirt designed to enhance workplace safety through multimodal monitoring and intelligent processing. The system incorporates a multitude of sensors for the comprehensive collection of data, including physiological sensors for the monitoring of vital signs such as heart rate and respiratory rate, as well as sensors for the capture of the worker’s movement and activity levels. This combination enables a comprehensive and detailed understanding of the worker’s state. The true intellectual capability of the SenseRisc system is manifested in its onboard software, which processes the collected data in real-time. The intelligent software platform is capable of analyzing and correlating the diverse data streams in order to identify potential health and safety risks that may arise during work activities. The system introduces the Preventive Risk Index (PRI), an innovative metric that synthesizes physiological and motion data to evaluate the risk levels associated with different working conditions. This novel indicator represents a significant advancement over previous studies, which typically focused on a singular aspect of health monitoring.

The efficacy of the SenseRisc system was substantiated in a laboratory setting, wherein eight healthy subjects engaged in a physical exercise protocol. This validation process evidenced the system’s capacity to accurately monitor physiological and motor parameters and to effectively determine the associated PRI. The development and integration of intelligent software into the wearable platform distinguish the SenseRisc system as a truly intelligent solution, providing a proactive approach to workplace safety and health monitoring.

The paper is structured as follows: Section 2 presents the overall architecture of the developed smart garment. Specifically, the instrumented shirt, the mobile application, and the intelligent software developed to manage data and estimate the PRI of the user are discussed. Section 3 describes the experiments carried out to test the smart garment’s performance. Section 4 discusses the obtained results and Section 5 outlines the conclusions of the present work and future works.

## 2. Materials and methods

**Figure 1.**
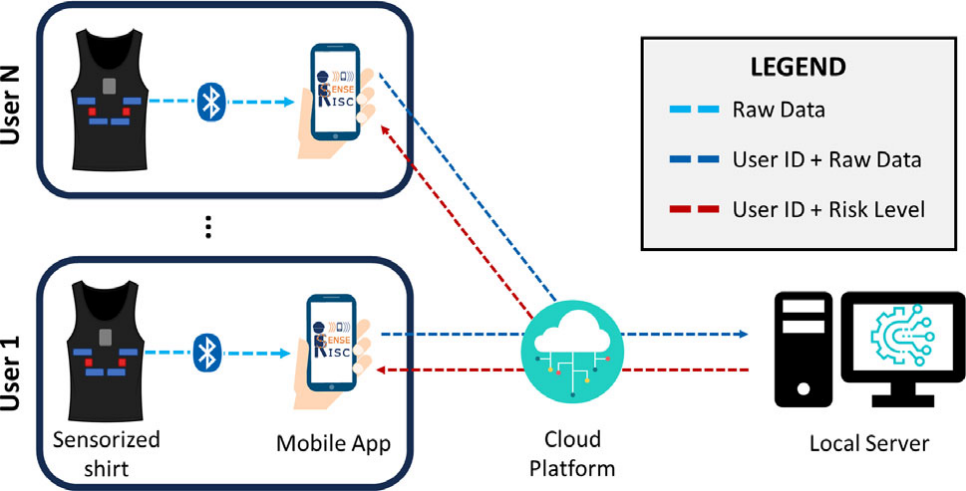
Architecture of the proposed instrumented smart shirt system.

The system architecture comprises three distinct subsystems: *I.* a physical shirt, that incorporates sensors for monitoring respiratory and cardiac functions and measuring the amount of the worker motion, *II.* a mobile application that is capable of collecting data locally and transferring it to a cloud platform, *III.* a local server that enables a bio-operative algorithm to assess the user PRI estimation for prevention and therefore identify potential health and safety hazards associated with occupational activities. Figure 1 provides an overview of the architectural configuration of the proposed system.

Each user is uniquely given an ID on the Mobile APP that unambiguously connects with the set of sensors on the smart instrumented shirt to monitor multi-modal parameters. Monitoring takes place in real-time during work activities via Bluetooth communication established between the application and the instrumented shirt, allowing the smartphone to receive raw data directly from the embedded electronics. The system is specifically designed so that the sensors communicate with the mobile phone using a Bluetooth Low Energy (BLE) connection, which is well suited to wearable applications due to its low power consumption and reliable short-range communication. In typical use, the distance between the shirt and the smartphone remains within 1 m, ensuring a stable connection. However, BLE theoretically supports ranges of up to 100 min open, unobstructed environments, while in real-world conditions that may include walls, metal structures, or electromagnetic interference, the range is typically reduced to 10–30 m. The use of BLE was primarily driven by the need for energy efficiency, as it minimizes battery consumption and ensures the system can operate continuously throughout an 8-hour shift. This design choice enables seamless, real-time data transmission without compromising the wearability or autonomy of the smart garment.

The collected raw data, along with the identifier of the user, are transmitted to a centralized cloud platform. This cloud-based approach offers scalability, accessibility, and data storage capabilities. It allows for secure data sharing and retrieval from anywhere, making it an excellent choice for a distributed workforce or remote monitoring scenarios. All data, each associated with its timestamp, are securely stored within the cloud platform, ensuring data integrity and easy access for authorized users.

The PRI associated with the worker is calculated by an intelligent algorithm that processes and fuses data coming from the multimodal monitoring system stored in the cloud on a local server. A Fuzzy Logic model is used to study the set of physiological and movement parameters to assess the degree of fatigue of the individual worker and return the baseline PRI. The use of the local server ensures timely computational operations, which reduce latency periods and provide real-time feedback to workers. The PRI is then sent back to each worker and displayed on the smartphone application.

### 2.1. Instrumented shirt

**Figure 2.**
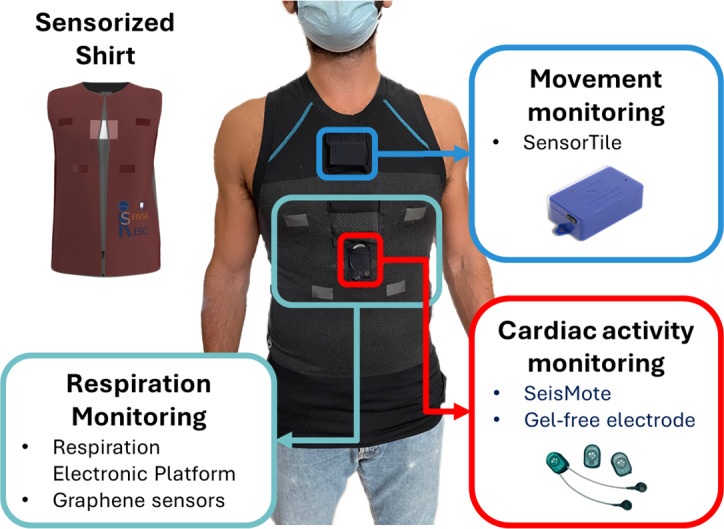
Representative user wearing the instrumented shirt and detail of each sensor system.

**Figure 3.**
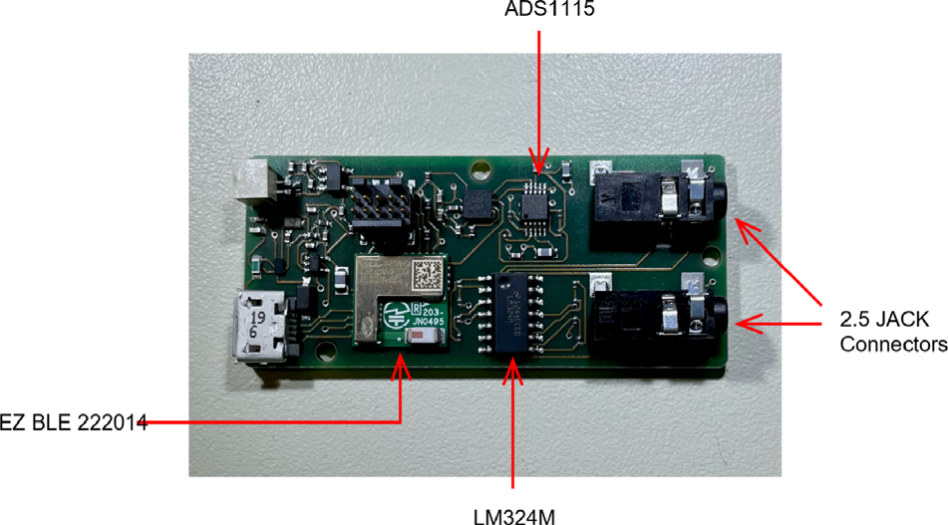
Respiration electronic platform (REP).

The SenseRisc solution is based on a commercial T-shirt integrating different sensors monitoring multimodal parameters. Figure 2 shows a representative user wearing the sensorized shirt detailing all the integrated systems for monitoring. More in detail, the SenseRisc system integrates monitoring systems for respiration, electrocardiogram (ECG), and movement. The respiration monitoring system comprises a Respiration Electronic Platform (REP) designed to receive raw signals collected from piezoresistive sensors, see Figure 3. These sensors are developed using a polymeric paint loaded with graphene nanoplates. The Seismote device was selected to acquire ECG signals, along with gel-free electrodes integrated into the inner layer of the shirt (further details are provided in Section 2.1.2). Finally, movement monitoring is achieved through the use of the SensorTile inertial platform. The following subsections will give details of the individual modules integrated into the SenseRisc system (technical information can be found in Section 2.1.3).

#### 2.1.1. Respiration monitoring

The respiratory monitoring system consists of four piezoresistive sensors, developed using polymeric paint loaded with graphene nanoplates. These sensors are printed on fabric employing a screen-printing technique, functioning as the piezoresistive strain gauges, as illustrated in Marra et al. (2021). The system is integrated with dedicated electronics, connected through a harness featuring conductive textile wires. The Respiration Electronic Platform (REP), reported in Figure 3, employs a Bluetooth Low Energy module (BLE, i.e., EZ-BLE 220124) that integrates an M0+ microcontroller (Lanata et al., 2020). This module is equipped with SPI and I2C serial communication channels for sensor communication. The Bluetooth module communicates in I2C with the Analog-to-Digital 16-bit ADC multichannel converter, which converts to digital values the analog voltage of the 4 analog channels corresponding to the sensors for respiratory signal monitoring. The textile strain gauges on the garment are connected to the electronic device through the three-pin 2.5 Jack Connectors.

Each respiratory sensor is acquired through a dedicated Howland circuit configuration (Maundy et al., 2019). It generates a constant current source passing through the respiratory sensor and providing a voltage drop proportional to the strain applied by the body to the sensors. A 3.7 V LiPo battery powers the electronic device. The respiratory sensors are acquired at a sampling frequency of 50 Hz.

#### 2.1.2. Cardiac activity monitoring

The integration of the ECG device within the inner lining of the smart shirt, leveraging the SeisMote platform and gel-free electrode embedded in the inner side of the smart shirt (Di Rienzo et al., 2020), represents a seamless fusion of cutting-edge technology and wearable design. This innovative wearable system, tailored for comprehensive cardiovascular monitoring in various settings, offers a unique combination of versatility and real-time capabilities. SeisMote utilizes a tailored low-power transmission protocol, ensuring the simultaneous acquisition of detailed data from up to 12 nodes, providing a comprehensive assessment of 36 signals at a high frequency of 200 Hz. This protocol guarantees precise time synchronization among nodes, minimizing jitter to less than 0.2 ms, fostering reliable and accurate data collection. For simplified applications requiring a single node, SeisMote supports Bluetooth Low Energy (BLE) protocol. Moreover, the system accommodates offline data collection, enhancing its flexibility. With an impressive battery life exceeding 16 hours on a single charge, SeisMote integration into the smart shirt reinforces its potential for prolonged, uninterrupted cardiovascular monitoring, making it an ideal choice for continuous health assessment in daily life.

#### 2.1.3. Movement monitoring

A SensorTile multisensor platform, manufactured by STMicroelectronics, was included in the SenseRisc system. Indeed, the SensorTile device includes a magneto-inertial measurement unit (Lapresa et al., 2022) and sensing units for the assessment of the environmental temperature, pressure, and humidity. The SensorTile platform lends itself well to the application of interest due to its small size, the presence of a Bluetooth Low Energy (BLE) module that enables data logging, and setting the acquisition frequency. This multisensor platform is placed in a dedicated pocket of the shirt on the chest of the worker (Di Tocco et al., 2022). SensorTile gathers information at 100 Hz and sends it via Bluetooth to the smartphone application.

### 2.2. Signal analysis and intelligent software module

The Intelligent software is the module that is in charge of retrieving the raw data acquired by the instrumented shirt, processing the raw data to obtain physiological and motion parameters of interest (that is the RR, the HR, and AL), and estimating the user PRI.

The first action performed by the intelligent software is downloading the raw data stored in the cloud. A query is designed to retrieve and parse documents uploaded by the app in the last 30 s to interpret the raw data.

The four respiratory signals are sampled at a sampling rate of 50 Hz. A third-order Butterworth band-pass filter is firstly applied to all the signals to exclude slow signal fluctuations not associated with respiratory movements (low cut-off frequency at 0.05 Hz) and remove frequency components beyond the respiration bandwidth (high cut-off frequency at 2 Hz) (Di Tocco et al., 2020). Given the availability of four signals extracted from the four piezoresistive sensors, we adopted a frequency-based strategy to determine a single respiratory rate (RR) value per instant (Massaroni et al., 2019). This method initially involves segmenting the signals into windows of appropriate temporal duration (Massaroni et al., 2023), then the selection of the sensor that provides the most relevant information in that time window, and finally the estimation of the respiratory rate (RR) from the signal recorded by that sensor. Hence, the filtered signals are segmented into 30 s length windows ( 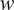 ), without overlap. For each 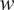 , the power spectral density (PSD) of the four signals is computed by using Welch’s overlapped segment averaging estimator, with an overlap value of 50% between segments. The signal with the highest power spectrum is the one selected for estimating RR in the 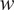 th window (Di Tocco et al., 2021). Considering that the maximum frequency peak of the PSD spectrum identifies the periodicity of the signal, the highest peak of the selected sensor PSD is considered to estimate the average RR (in bpm) of the 30 s segment as presented in Equation (1).(1)

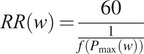




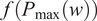
 is the frequency associated with the highest peak of the power spectrum of the selected sensor.

The ECG signal is sampled at a frame rate of 200 Hz. Starting from the raw signal, the Pan-Tompkins algorithm is applied to detect the QRS complex (Fariha et al., 2020). It consists of a sequence of filters that enhance the frequency content of the electrical activity of the heart and remove the background noise. Finally, the algorithm includes rectification of the signal to amplify the QRS complex to make it easy to identify. When R-peaks are identified in the ECG signal, it is possible to compute the inter-beat interval ( 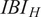 ). The 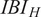 , commonly referred to as the tachogram, is the time duration between successive R-peaks. Utilizing the 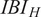 signal as a foundation, it becomes feasible to derive the instantaneous heart rate (HR) using Equation (2), measured in beats per minute [bpm].(2)

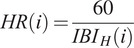

 where 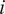 indicates the 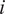 th detected R-peak.

The accelerations measured along the three axes of the accelerometer mounted inside the Seismote device can provide synthetic information about an individual’s activity level (AL) as reported in Equation (3)
(3)

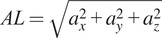




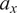
 , 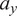 , and 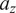 are the acceleration computed along the 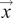 , 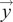 , and 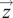 Cartesian axes, respectively. AL represents the overall intensity of movement captured by the accelerometer, regardless of the direction of the movement.

Given RR, HR, and AL, a Fuzzy Logic model was developed to handle variability inherent in physiological and motion-related data and assess the PRI of the worker. The essential steps required in the definition of Fuzzy Logic systems are the definition of the input and output Membership Functions (MFs), and the definition of the fuzzy rules.

MFs were then modeled for each input parameter, that is, RR, HR, and AL, and for the output PRI to translate the parameters extracted from the data monitored by the T-shirt and the returned indicator into linguistic variables representing the amount of activation of the specific input and output (Tamantini et al., 2024). In a Fuzzy Logic approach, linguistic variables translate complex, quantitative data into intuitive and easily interpretable qualitative descriptions. Terms like “low,” “normal,” and “high” can be used to model the variability of the physiological parameters. These terms correspond to numerical ranges defined by MFs, indicating the degree to which a value belongs to each category. This enables the Fuzzy Logic system to apply rules that emulate human reasoning. Specifically, the proposed model exploits three, four, and two trapezoidal membership functions for RR, HR, and AL, respectively. Among all the possible shapes that can be used to design the MFs, trapezoidal membership functions were selected due to their flexibility and ability to capture gradual transitions in linguistic variables. Furthermore, the shape of the trapezoid corresponds to a clear linguistic interpretation, allowing a simpler understanding and interpretation of the membership degrees associated with different input values. The general equation describing the trapezoidal MF is reported in Equation (4).(4)

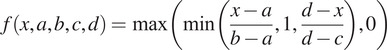




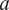
 and 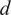 locate the feet of the trapezoid, 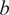 and 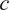 locate the shoulders of the trapezoid MF, and 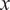 is the input parameter.

Moreover, it is worth observing that a different number of MFs for each input parameter was used in order to provide an adequate representation of the linguistic variables associated with the physiological and motion parameters. More in detail, for each input and output, specific linguistic variables were introduced.

For RR, the three MFs reflect the general patterns observed in respiratory rates, considering the typical ranges for low RR (**L**), normal (**N**), and high RR (**H**). The activation levels for respiratory rate (RR) were defined based on clinical thresholds for abnormal breathing patterns, specifically bradypnea and tachypnea. A respiratory rate is considered normal when it falls between 12 and 16 breaths per minute. Values below 12 bpm are classified as bradypnea, indicating abnormally slow breathing, while sustained increases above the normal range characterize tachypnea, a condition associated with abnormally rapid breathing and potential clinical concern (Hill and Annesley, 2020).

For HR, four activation levels, Low (L), Normal (N), Mid-High (MH), and High (H), were defined using trapezoidal membership functions. These ranges were selected to reflect both clinical thresholds and physiological responses to physical exertion. Specifically, HR values below 60 bpm (L) may indicate bradycardia, while values between 60 and 100 bpm (N) are generally considered normal resting ranges in healthy adults. The Mid-High (MH) and High (H) categories capture progressive cardiovascular strain, with HR exceeding 100 bpm reflecting increasing workload and values above 160 bpm indicating intense physical effort (Cook et al., 2006; Nanchen, 2018).

Finally, two membership functions distinguished between low (**L**) and high (**H**) AL, providing a concise representation of the level of physical activity of the participants.

**Figure 4.**
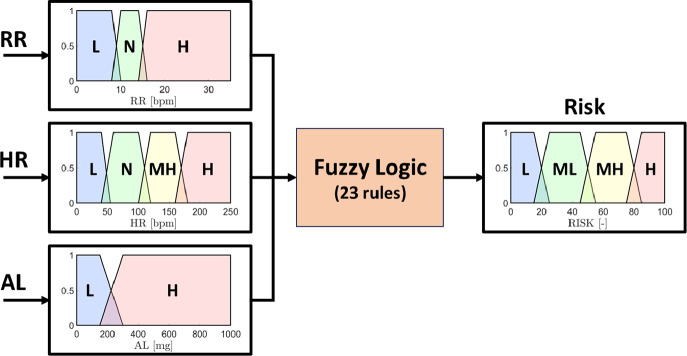
Fuzzy Logic Model implemented into the instrumented intelligent shirt for Preventive Risk Index estimation.

On the output side of the Fuzzy Logic inference model, the PRI returned by the model was modeled as four MFs resembling low (**L**), mid-low (**ML**), mid-high (**MH**), and high (**H**) conditions. Table 1 reports the list of the trapezoidal coeffectives per MF adopted in the Fuzzy Logic model. A graphical representation of the Fuzzy Logic Model is depicted in Figure 4.

**Table 1.** Parameters of the trapezoidal MFs adopted in the Fuzzy Logic Model

		a	b	c	d
RR [bpm]	L	0	0	8	10
	N	8	10	14	16
	H	14	16	35	35
HR [bpm]	L	0	0	40	60
	N	40	60	100	120
	MH	100	120	160	180
	H	160	180	250	250
AL [mg]	L	0	0	150	300
	H	150	300	1,000	1,000
PRI [−]	L	0	0	15	25
	ML	15	25	45	55
	MH	45	55	75	85
	H	75	85	100	100

Given the linguistic representation of the input parameters provided by the MFs, a fuzzy rule set composed of 23 rules was implemented, taking inspiration from literature pieces of evidence (Tamantini et al., 2024).

**Figure 5.**
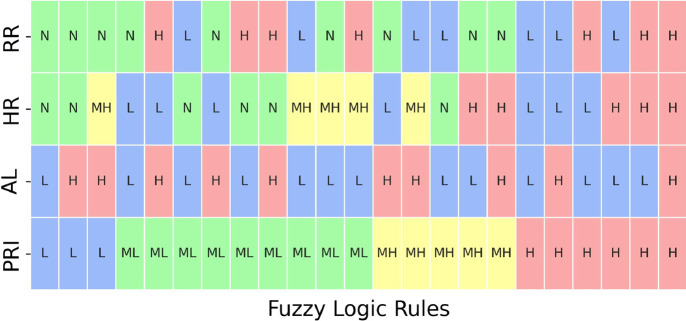
Graphical representation of the rules implemented in the Fuzzy Logic model for PRI estimation.

Each rule was developed by combining the activation levels of the monitored parameters, which were defined based on physiological thresholds and known indicators of physical stress. The resulting combinations were then associated with a specific PRI level. For example, conditions where RR and HR remain within normal ranges typically correspond to low-risk scenarios, even with moderate activity. Conversely, combinations of abnormal HR or RR values, such as bradycardia, tachycardia, or irregular breathing, especially when combined with high activity, are mapped to higher risk levels. This approach allows the system to provide an interpretable and physiologically consistent assessment of the worker’s condition. The full set of rules implemented in the fuzzy logic model is shown visually in Figure 5, supporting a continuous and real-time estimation of PRI based on multimodal physiological and motion signals.

### 2.3. Mobile application

A smartphone Android application (App) is developed to perform local data viewing, cloud communication, and activation/deactivation of nodes (i.e., SensorTile, REP, and Seismote). Sensor data can be stored both locally and in the cloud. The smartphone acts as the central node of the network, enabling control of the external nodes. Through the App, users can manage data transmission for each node and sensor individually, as well as start or stop the data acquisition process. Additionally, the App allows for data synchronization across multiple mobile devices and makes it accessible for back-end operations. The App architecture is based on the communication among multiple GATT Servers, allowing simultaneous node connection and individual control over the data streaming that the GATT Servers expose to the Client through the supported Bluetooth Low Energy Channels (BLE-Chs). During the process of data acquisition, records are saved locally on the smartphone and in real time sent to the cloud. The mobile application uniquely connects to the set of nodes that make up the system; these can be manually disabled if necessary. On the graphical user interface (GUI) the status of each node “Device Info” is shown. Once the connection is established, BLE in the “Characteristic Control” section displays the sensor feature and data is streamed.

**Figure 6.**
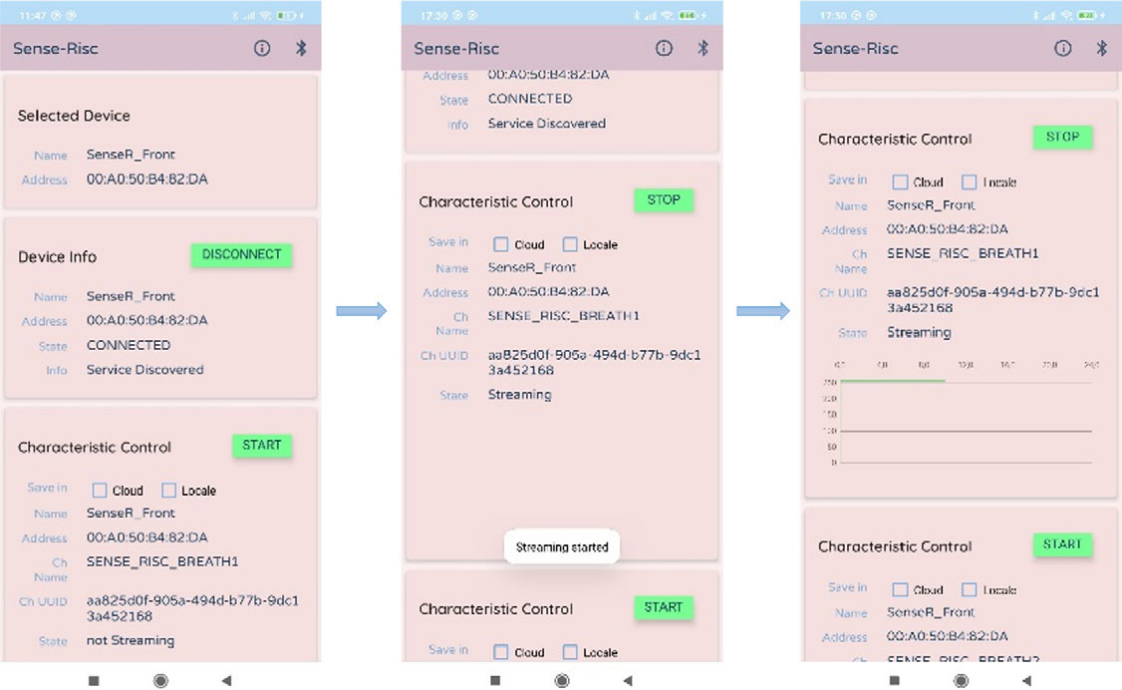
Sequence of UI updates when activating sensor notifications.

**Figure 7.**
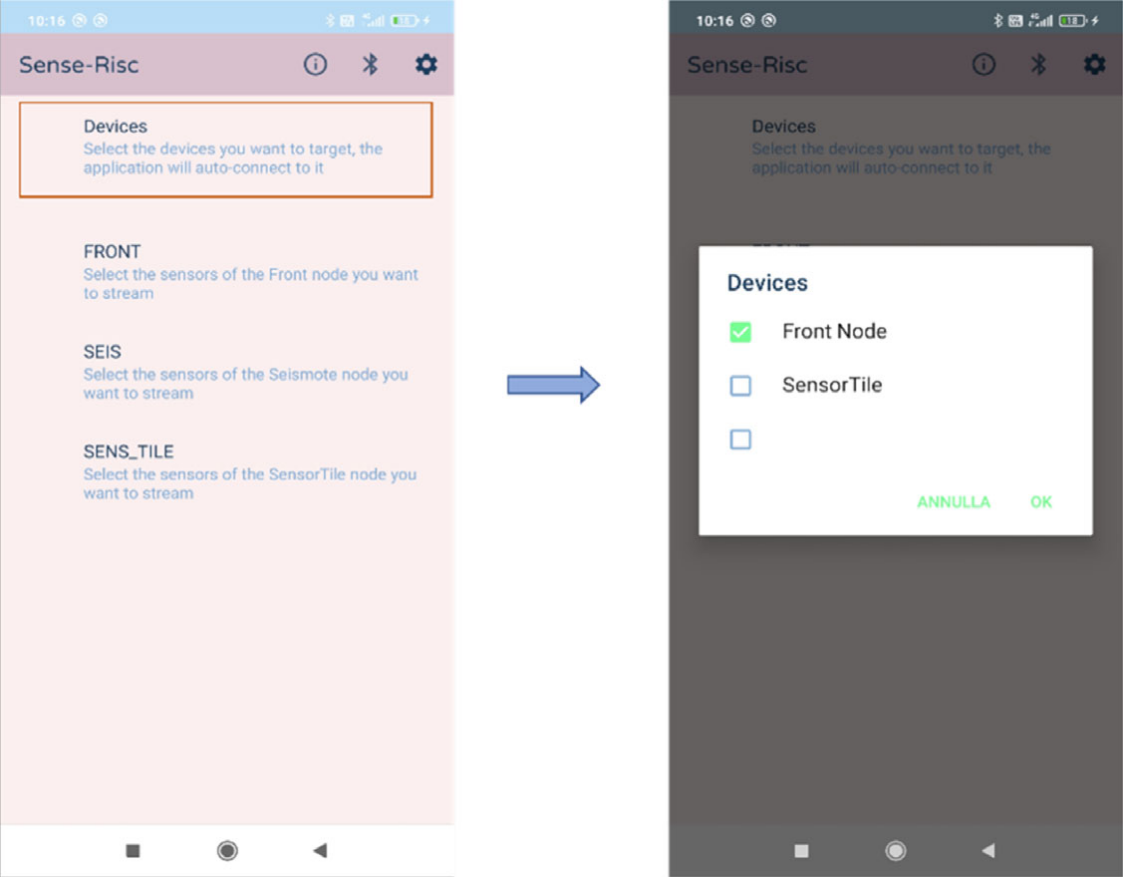
Node selection mechanism.

Figure 6 shows the sequence of updates to the UI that occurs when the user activates the notification on the sensor feature. When streaming is activated, the graph within the section will be updated whenever new data is notified to the node. Each section for streaming control contains two checkboxes, the former to enable local data storage and the latter to enable cloud storage (see Figure 7).

Data is fully synchronized at the instant the last sensor starts streaming, and the application starts collecting the data from the different sensors into separate data structures. Each structure has a length in bytes ( 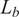 ) that can be computed as in Equation (5).(5)

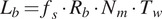

where 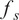 is the sampling rate of the sensor, 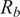 is the size in bytes of the individual sensor sample, and 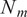 is the number of measurements in the characteristic acquired in a time window of 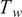 seconds.

**Figure 8.**
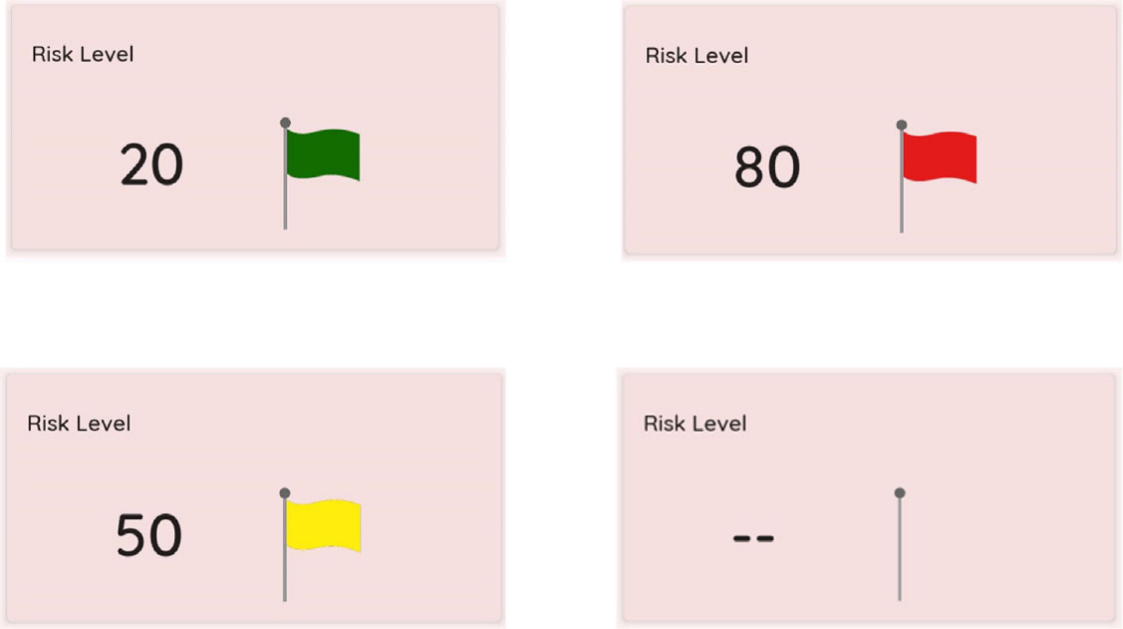
Mobile App displaying the estimated Preventive Risk Index of the user.

The app receives a notification whenever a new file containing the PRI value is published to the cloud (see Section 2.2 for PRI computation). Specifically, if the PRI has been published to the cloud in the last 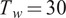 s, the PRI is shown on the app UI, and a flag colored green, yellow, or red depending on the value taken by the LdR, otherwise, only the flagpole is shown. Figure 8 shows the graphical section of the Mobile App that displays the PRI.

## 3. Experimental validation

To assess the capabilities of the developed instrumented shirt in monitoring users’ physiological and motion parameters and estimating the PRI, an experimental session was designed to measure the performance of the developed smart garment in a laboratory setting, before moving to a workplace. For this purpose, the experimental protocol was designed to generate relevant variations in the subject’s physical conditions (up to physical stress) and assess whether the sensors integrated into the smart shirt are able to detect different conditions of the subject. At the same time, the PRI should reflect the different states of the participants during the administered protocol.

Eight males ( 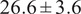 y.o.) were enrolled in this experiment. All the participants were healthy and had never suffered from any disease that might be contraindicated for the current experiment. They signed a written consent to be enrolled in the study before participating and, afterward, they were instructed to wear the instrumented shirt.

The participants were asked to perform three different activities during the experimental protocol:

- Lifting a load (LIFT): participants started exerting by repetitively lifting from the ground a 3 kg box for 2 min. They were instructed to perform the lifting procedure in a standardized manner.
- Moving a load (MOVE): the participants were asked to manually carry the same 3 kg box while walking at a speed of approximately 1 m/s. To ensure that the walking speed was consistent, a metronome was used to regulate the cadence of their steps.
- Intense physical activity (PHYSICAL): participants performed jumping jacks for 2 minutes. Exploiting this highly dynamic exercise, it was possible to induce an intense level of physical exertion in the enrolled volunteers.

The physically stressful activities were administered to assess whether the shirt could capture different AL and physiological responses in the participants. Each activity was performed for 2 minutes.

The complete protocol involved repeating the sequence LIFT-MOVE-PHYSICAL followed by a second round of LIFT-MOVE. This repetition was designed to evaluate the recovery dynamics of physiological parameters after intense activity, providing deeper insights into the shirt’s capability to monitor changes over time and under varied physical demands.

**Figure 9.**
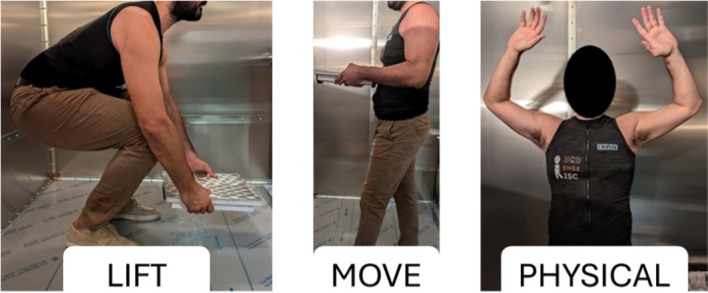
A representative participant performing the three activities included in the experimental protocol.

A picture depicting a representative subject during the protocol execution is reported in Figure 9.

Moreover, the Mann–Whitney test was employed to determine whether significant differences exist in monitored parameters and PRI between successive activities. This non-parametric test was selected due to its capability to deal with non-normally distributed data. The significance level was set at 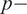 value 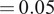 .

To assess the subjective perception of the intelligent shirt, the participants were asked to evaluate the usability of the system. In particular, the System Usability Scale (SUS) provides a quick and reliable measure of the usability of any device. Scores ranging from 0 to 5 collected from 10 different statements were combined and transformed into a single usability score, ranging from 0 to 100. The higher the score, the higher the perceived usability. Any system has a satisfactory usability score if it achieves a SUS score 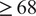 (Bangor et al., 2009).

Moreover, weight (W), breathability (BR), shape and size (SS), skin sensitivity (SE), adjustment (AD), and mobility (M) represent other factors that are paramount during wearable systems evaluation. Weight is crucial for comfort, as a heavy device can cause discomfort during prolonged use. The breathability of the fabric is important to prevent excessive sweating and discomfort. The shape and size should fit the body without causing pressure or compression. Skin sensitivity is vital, as materials can potentially cause irritation or allergies. Adjustability ensures a personalized fit, contributing to overall comfort. Finally, good mobility allows the wearable device to move with the body, enhancing comfort during use. These were assessed employing an ad hoc questionnaire on a Likert scale ranging from 1 to 7 representing “strongly disagree” to “strongly agree,” respectively. The list of administered questions is reported in the following:

- **W1**: The instrumented shirt was light and comfortable to wear.
- **W2**: The instrumented shirt was heavy and bulky.
- **BR1**: The instrumented shirt was breathable and promoted ventilation.
- **BR2**: The instrumented shirt caused excessive sweating and a lack of ventilation.
- **SS1**: The instrumented shirt fit my body shape well.
- **SS2**: The instrumented shirt was either too tight or too loose.
- **SE1**: The instrumented shirt did not irritate my skin.
- **SE2**: The instrumented shirt caused irritation or discomfort to my skin.
- **AD1**: It was possible to adjust the sensor T-shirt to suit my needs.
- **AD2**: The sensor T-shirt did not allow adequate adjustment.
- **M1**: The instrumented shirt allowed fluid and unrestricted movements.
- **M2**: The instrumented shirt restricted my freedom of movement.

As evident, out of the 12 questions, the even-numbered questions are reverse-scored. This means that to combine the results per factor, the scores for these questions need to be inverted. The items related to the same factors were averaged per participant to provide a synthetic representation of the score obtained.

## 4. Results and discussions

**Figure 10.**
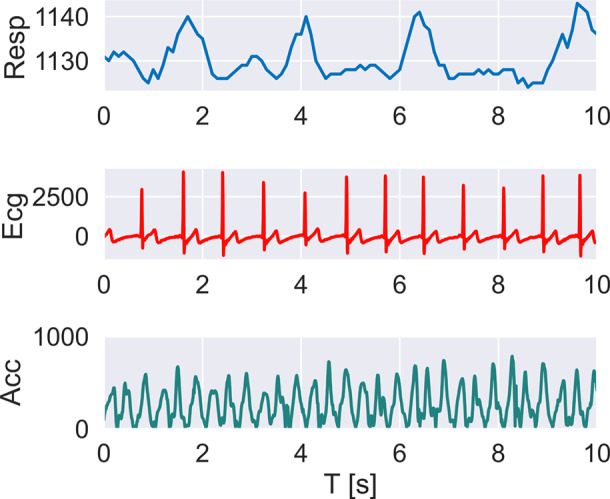
The raw signals of physiological parameters and the norm of the acceleration collected with the instrumented smart shirt of a representative subject during the execution of the lifting task are presented. From top to bottom, the respiration waveform (Resp), the ECG, and the norm of the acceleration (Acc) collected from the REP, the Seismote, and the SensorTile are reported.

Figure 10 displays the raw physiological signals, that is, the respiration waveform (Resp) and the ECG, along with the norm of the acceleration (Acc) captured by the intelligent shirt of a representative subject in 10 s.

It is worth observing that the fabric-printed sensor for respiratory signal monitoring is capable of capturing both the expansions and compressions of the rib cage during the lung ventilation process. The ECG signal measured by the wearable system is also very clear and readable, especially its R complex, fundamental for calculating the HR of the users.

**Figure 11.**
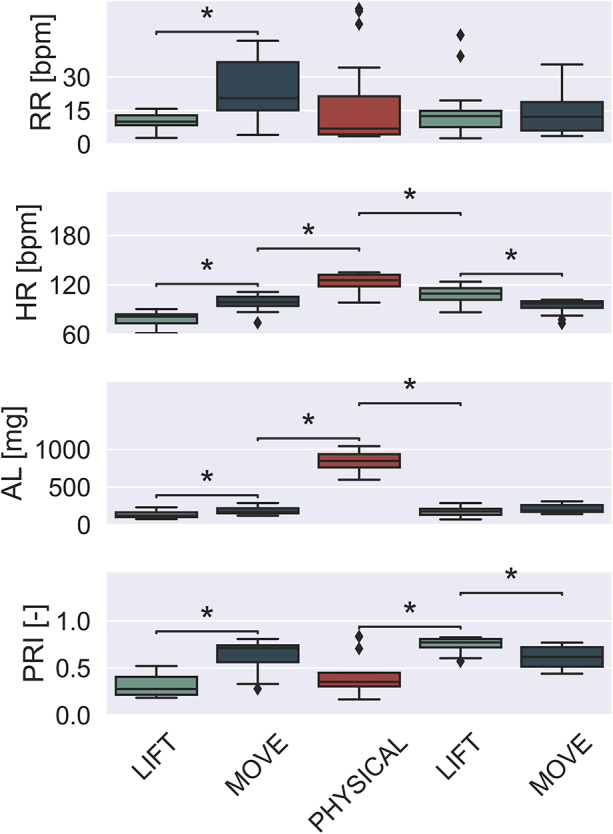
Boxplot of monitored physiological parameters, that is, the Respiratory Rate (RR) and the Heart Rate (HR), along with the amount of movement expressed as Activity Level (AS) and estimated Preventive Risk Index (PRI), averaged across the 8 enrolled participants, stratified for each performed activity. The statistical test was applied between one activity and the subsequent one. * denotes a statistical difference ( 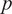 -value 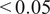 ).

Figure 11 depicts the distribution of the PRI (PRI) and other monitored parameters, that is, RR, HR, and AL, across various activities obtained from the eight enrolled subjects. The boxplot compares the central tendency, variability, and outliers in the PRI and related parameters across activities. The median value is shown by the central line, the interquartile range (IQR) is indicated by the box edges, and whiskers extend to 1.5 times the IQR. Points beyond the whiskers are outliers of the distribution.

As the physical activities performed by the enrolled subjects varied, the monitored physiological and motion parameters showed changes over time. The RR showed an increasing trend in the early stages of physical fatigue during the first repetition of the LIFT and MOVE tasks, where peaks of 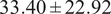 bpm were reached. As the intensity of the physical activity increases, during the execution of the jumping jacks, physical fatigue becomes evident, reflected in a decrease in the respiratory rate (Meng et al., 2014; Tamantini et al., 2021). During the second series of LIFT and MOVE exercises, the RR stabilizes around 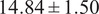 bpm without showing any other significant changes.

Conversely, cardiac activity reflects the phenomena of fatigue and relaxation towards less strenuous activities. Increasing the intensity of the physical activity shows how the HR goes from 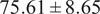 bpm, at the beginning of the experimental protocol, to 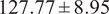 bpm at the end of the execution of the PHYSICAL task. When subjects start again with the LIFT and MOVE, less physically demanding than jumping jacks, a physiological recovery of HR is observed.

AL parameter, calculated from the information of the accelerometer integrated into the smart shirt, reflects the intensity of the activities performed: 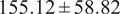 , 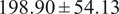 , and 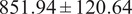 mg are the AL calculated for lifting, moving, and physical tasks, respectively.

Finally, the PRI reflects the overall state of the user taking into account the activity he or she is performing. At the beginning of the protocol, during the LIFT activity, the PRI is 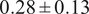 . Continuing the execution of the activities administered to the participants, the altered physiological state (in terms of an increased RR) and motor condition are reflected in the PRI proposed in the SenseRisc system becoming 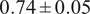 at the end of the MOVE activity. When the physical activity becomes significantly more intense, PRI drops again to 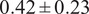 . During physically demanding activities, if the SenseRisc user is in good health, the intelligent algorithm assigns a low PRI value. Subsequently, when the user returns to perform low-impacting activities, the PRI is higher with respect to the one observed in the first repetition. During the execution of low-impact activities, the physiological state of the user is expected to be comparable to a resting condition. Conversely, the experimental protocol allows observing the unwinding of activities at low physical load with the participant in a condition of high physiological stress reflected in a PRI of 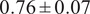 at the beginning of the second LIFT activity.

**Figure 12.**
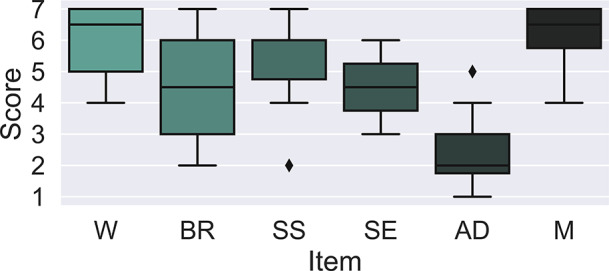
Lickert scores assessing the intelligent shirt weight (W), breathability (BR), shape and size (SS), skin sensitivity (SE), adjustment (AD), and mobility (M).

From a subjective perspective, the intelligent shirt achieved a SUS score of 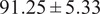 . This score represents a strong vote of confidence from the participants regarding the usability of the SenseRisc system. This favorable usability assessment ensures good usability in operative scenarios. Moreover, Figure 12 shows the scores obtained from the administration of the 7-point Likert scale specifically assessing the other usability aspects of the intelligent shirt.

The participants rated the weight of the smart shirt with an average score of 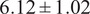 . Participants found the weight of the shirt to be relatively comfortable. The breathability of the fabric used in the smart shirt received an average rating of 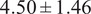 . This score suggests that some participants found the wearable system to be more breathable than others. The SS received an average rating of _s_, suggesting that, on average, participants were generally satisfied with its fit and dimensions. Providing customization options for fit could enhance user comfort further. Participants rated skin sensitivity concerning the smart shirt with an average score of 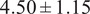 . This suggests that, on average, participants had moderate perceptions of skin sensitivity. Ensuring that the materials used in the shirt are hypoallergenic and comfortable for a broad range of users is essential. The AD feature received a lower average rating of 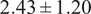 , suggesting that participants found the shirt adjustment less satisfactory on average. Improving the ease of adjustment could enhance overall comfort and usability. The mobility of the smart shirt received a favorable average rating of 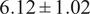 indicating that participants perceived the shirt as providing good mobility during use. This is a positive aspect as it contributes to the overall user experience.

In summary, the smart knit showed strengths in terms of weight and mobility. However, there is room for improvement in terms of perceived BR, AD, and SS. These results highlight the importance of considering user feedback and comfort when designing wearable technology. Indeed, it is necessary to adapt the smart knit to different body types, both for comfort and acceptability of the SenseRisc system, but also to ensure the correct detection of physiological parameters to be monitored by the sensors integrated into the intelligent shirt. It is of crucial importance to adapt this technology to different sizes in order to meet the builds of the target users.

The intelligent shirt has proven itself as a reliable tool for capturing physiological processes and motion data, even in physically challenging tasks. Thanks to the intelligent software, this raw data is processed, allowing for real-time PRI assessment. Moreover, the data exchanged by the platform are always labeled with the specific identifier of the user. This means that the system can process data from multiple operators and return its estimated PRI to each. This aspect is paramount to understanding the power of the scalability of such a personal device. Finally, subjective evaluations have offered valuable insights into the acceptability and usability of the system. This approach underscores the potential of the SenseRisc system to significantly enhance workplace safety and the overall experience of users.

The specific physiological signals monitored by SenseRisc and the possibility of implementing additional on-board processing functions open the possibility of exploring the application of this system in other health-related monitoring scenarios, such as during hospital-based cardiorespiratory rehabilitation and/or home telerehabilitation. Furthermore, thanks to the modular nature of the system, it is possible to integrate additional sensors depending on the specific needs of the application scenario. This flexibility enables the platform to be adapted to monitor different types of risk factors beyond those related to physical exertion.

For example, in occupational settings involving chemical exposure or work in low-oxygen environments, additional modules, such as gas detectors or pulse oximeters, could be integrated into the smart garment. While the current version of SenseRisc focuses on monitoring risk factors associated with physiological overload (e.g., elevated heart and respiratory rate during physical activity), the architecture of the system is designed to support future expansions. This adaptability makes it suitable for a wide variety of use cases, allowing for tailored configurations that respond to the specific risks associated with different job roles or work environments.

It is important to note that, although the PRI proposed in this work is not a clinically validated metric, the system itself is not intended as a medical device. Rather, it serves as a technological solution aimed at improving workplace safety. Its primary goal is to provide a practical and interpretable indicator of physical strain, enabling the early identification of potentially risky conditions during work tasks, especially those involving fatigue or physiological overload that might go undetected by conventional safety assessments. The fuzzy logic-based PRI model maps multimodal physiological and motion signals into discrete risk levels using a rule-based framework derived from prior literature and physiological knowledge, ensuring both transparency and relevance in risk estimation.

Finally, to enable the transition of the SenseRisc system into real occupational settings, several steps are required. First, it is necessary to extend testing to a larger and more heterogeneous population of users, capturing inter-individual variability and the influence of different job roles and physical demands. Second, the system must be adapted for long-term wear, ensuring comfort, durability, and sensor stability over full work shifts and in diverse environmental conditions. Third, it will be essential to engage with health and safety officers and companies to define deployment protocols that integrate seamlessly with existing workflows. Finally, additional testing may be needed to verify safety, data privacy compliance, and effectiveness in detecting high-risk situations before full-scale deployment in industrial scenarios.

## 5. Conclusion

This paper presented the SenseRisc system, an instrumented intelligent shirt designed to prevent risks in workplaces. The proposed system represents a significant advancement in the field of wearable technology, showcasing its potential to integrate a diverse array of sensors directly inside the garments of the worker. Respiratory waveforms, ECG, and user acceleration are constantly monitored by the SenseRisc shirt. Moreover, the smartphone application, presented in this paper, eases the data collection and sharing with a cloud system and provides the PRI of the user on feedback. With the presence of an intelligent algorithm, this system can monitor and process in real-time the physiological and motor parameters of workers undergoing physically stressful activities.

The results presented in this work highlight the capability of the intelligent shirt to measure physiological signals with a high degree of fidelity even during the execution of physically demanding tasks. The intelligent software then processes the raw data collected to retrieve the PRI of the user in real time. The experiment carried out enrolling eight healthy participants showcased the capability of the proposed software to map imprecise and highly variable physiological parameters into a target variable estimating the risk condition in which the worker is. The intelligent shirt was also evaluated from a subjective point of view, providing a comprehensive view of the acceptability and usability of such a system.

Future work will focus on the optimization and scaling of the SenseRisc system for deployment in real construction sites, where we plan to carry out a broader validation and usability assessment under actual working conditions with several users simultaneously. In parallel, efforts will also be dedicated to the development of different shirt sizes to accommodate a range of body types better and ensure comfort and wearability across diverse user populations.

## Data Availability

The data that support the findings of this study are available from the corresponding author, C.T., upon reasonable request.
